# Visible-Light-Driven Degradation of Chloramphenicol Using CeO_2_ Nanoparticles Prepared by a Supercritical CO_2_ Route: A Proof of Concept

**DOI:** 10.3390/nano15020102

**Published:** 2025-01-10

**Authors:** Maria Chiara Iannaco, Antonietta Mancuso, Stefania Mottola, Andrea Pipolo, Vincenzo Vaiano, Iolanda De Marco

**Affiliations:** Department of Industrial Engineering, University of Salerno, Via Giovanni Paolo II 132, 84084 Fisciano, Salerno, Italy; miannaco@unisa.it (M.C.I.); anmancuso@unisa.it (A.M.); smottola@unisa.it (S.M.); apipolo@unisa.it (A.P.); idemarco@unisa.it (I.D.M.)

**Keywords:** supercritical CO_2_ route, Ce(acac)_3_ nanoparticles, CeO_2_, photocatalysis, visible light, chloramphenicol

## Abstract

Recently, the extensive use of antibiotics has unavoidably resulted in the discharge of significant quantities of these drugs into the environment, causing contamination and fostering antibiotic resistance. Among various approaches employed to tackle this problem, heterogeneous photocatalysis has emerged as a technique for antibiotic degradation. This study explores the potential of CeO_2_ as a photocatalyst for the degradation of chloramphenicol. Supercritical antisolvent (SAS) processing was successfully employed to synthesize photocatalyst precursor nanoparticles. After thermal annealing, the CeO_2_ samples were characterized through UV–Vis diffuse reflectance spectroscopy to evaluate the band gap energy values. Raman and FT-IR spectroscopy confirmed the presence of oxygen vacancies in the CeO_2_ lattice. During photocatalytic experiments, the CeO_2_ derived from the SAS-processed precursor exhibited superior photocatalytic performance compared to the catalyst synthesized from the non-micronized precursor. Various annealing temperatures were employed to tune the oxygen vacancy of CeO_2_. Furthermore, the impact of catalyst dosage and chloramphenicol concentration was investigated. Under optimal reaction conditions (25 mg L^−1^ chloramphenicol and 2.25 g L^−1^ catalyst dosage), a degradation efficiency of 64% was achieved. Finally, to elucidate the degradation mechanism, different scavengers (EDTA, benzoquinone, and isopropyl alcohol) were utilized, revealing that the superoxide radical is the primary species responsible for chloramphenicol degradation.

## 1. Introduction

Since their discovery, antibiotics have been used extensively to treat various bacterial infections in humans and animals. However, most antibiotic molecules are only partially broken down in the body, leading to residual compounds or metabolites that can enter wastewater treatment plants (WWTPs) [1,2,3]. However, wastewater treatment processes cannot fully eliminate or degrade these compounds. It was shown that therapeutic activities are responsible for the presence of antibiotic residues in the effluents of wastewater treatment plants, surface waters, groundwater, and water used for aquaculture. Antibiotic accumulation in the environment has led to the development of antibiotic-resistant bacteria. In fact, due to their poor biodegradability, antibiotics can persist in the environment and exhibit toxic properties toward microorganisms even at trace levels [4,5,6].

Chloramphenicol (CAP) is a broad-spectrum antibiotic that inhibits bacterial protein synthesis [7]. It’s bacteriostatic at lower concentrations and bactericidal at higher doses [8]. CAP is a versatile antibiotic effective against a wide range of bacterial infections in both humans and animals, including Gram-positive, Gram-negative, and anaerobic bacteria. Its notable stability and resistance to degradation pose a significant challenge for wastewater treatment plants in eliminating it from the environment [9]. As a result, it is frequently released into water bodies. Moreover, if treated wastewater or WWTP sludge is applied to agricultural lands, extensive farmland and nearby water sources may become heavily contaminated with CAP. Several methods have been devised to diminish or eliminate CAP levels in water bodies. These techniques encompass traditional approaches like flocculation, membrane filtration, coagulation, and biodegradation, as well as innovative methods like advanced oxidation processes, adsorption, and zero-valent iron [10,11,12,13,14].

Among these, heterogeneous photocatalysis has garnered significant attention [15]. Indeed, photocatalysis is widely used to remove organic pollutants from both industrial and municipal wastewater [16,17,18,19,20,21]. The main advantage of photocatalysis lies in its ability to completely mineralize organic contaminants, driven by reactive oxygen species such as hydroxyl radicals (·OH) and superoxide radicals (O_2_^.−^) at room temperature and atmospheric pressure [22,23]. Unlike the adsorption process, where contaminants are transferred to another medium, photocatalysis leads to complete degradation [24,25,26,27,28]. This not only reduces the need for secondary disposal methods but also employs ambient oxygen and water, making it a cost-effective technique. An effective photocatalyst possesses several essential characteristics. First, it should be photoactive to utilize visible or near UV light; in addition, it must be chemically inert and photostable to ensure long-lasting performance.

Over the years, the possibility of using photocatalysis for the degradation of chloramphenicol has been studied primarily by employing TiO_2_-based materials as photocatalysts [29,30,31,32]. However, due to its band gap, TiO_2_ has limited practicality, since it requires ultraviolet light for activation, which is only 3–5% of solar radiation [33,34,35,36]. Ongoing research efforts are focused on the development of highly efficient photocatalysts active in the visible region for the degradation of CAP [37,38,39,40]. In this perspective, CeO_2_ is a rare-earth oxide that can absorb visible light because of the strong reducibility of Ce^4+^ ions to Ce^3+^, which creates significant oxygen vacancies [41,42]. In addition, this high reducibility prevents electron–hole recombination [43,44]. As a result, it is thought to be among the most promising semiconductors available for photocatalytic applications [45,46,47]. The photocatalytic properties of CeO_2_ strongly depend on the size and shape of the nanoparticles. In this regard, intensive research has focused on controlling the surface area and morphology of synthesized CeO_2_ nanomaterials to optimize their performance [48,49,50]. Traditional methods widely utilized for the synthesis of heterogeneous photocatalysts exhibit several limitations, primarily related to the lack of control over catalyst morphology, size, and particle size distribution, which affect the photocatalyst performance. Supercritical carbon dioxide (scCO_2_)-assisted techniques have emerged as an alternative to overcome these challenges [51,52]. The critical review by Franco et al. [53] provides an in-depth analysis of the potential and the advantages associated with the use of supercritical carbon dioxide in the synthesis process of nanometric photocatalysts. Specifically, these advanced methods enable the synthesis of photocatalysts while minimizing reliance on toxic and environmentally harmful solvents. It is essential to highlight that the number of studies specifically focused on utilizing pure CeO_2_, devoid of dopants or coupled with other semiconductors, for the photocatalytic degradation of CAP under visible light is still scarce.

Therefore, this study aims to investigate the photocatalytic degradation of chloramphenicol using cerium oxide (CeO_2_) as a photocatalyst and to clarify the potential of using a micronized precursor for the synthesis of CeO_2_. Indeed, the supercritical antisolvent (SAS) process has been employed to obtain CeO_2_ precursor particles at the nanoscale [54]. The SAS process exploits the properties of carbon dioxide under supercritical conditions, i.e., density comparable to that of liquids and diffusivity of the order of magnitude of those of gases [55]. By appropriately selecting the organic solvent, temperature, pressure, concentration of the solute in solution, and the ratio between the carbon dioxide and liquid solution flow rates, it is possible to obtain microparticles or nanoparticles of the compound of interest [56]. In particular, in this work, the effect of micronization on the photocatalyst performance was evaluated. Subsequently, the degradation kinetics of chloramphenicol was investigated by varying different operating parameters (photocatalyst dosage and antibiotic concentrations). The relevance of the calcination temperature was also examined. This study must be intended as a proof-of-concept investigation to determine the feasibility of utilizing the SAS method in future research to develop suitably modified CeO_2_-based photocatalysts capable of activating under visible light conditions and able to enhance the water pollutants’ photodegradation performance.

## 2. Materials and Methods

### 2.1. Materials

Cerium (III) acetylacetonate hydrate (Ce(acac)_3_, CAS 206996-61-4) was purchased by Thermo Scientific Chemicals (Segrate, Italy). Sigma Aldrich supplied Chloramphenicol (CAS 56-75-7). Ethylic alcohol (purity 99.9%) was purchased from Carlo Erba Reagenti (Cornaredo, Italy). CO_2_ at 99% purity was purchased by Morlando Group s.r.l. (Naples, Italy).

### 2.2. Micronization of Ce(acac)_3_

**Figure 1:**
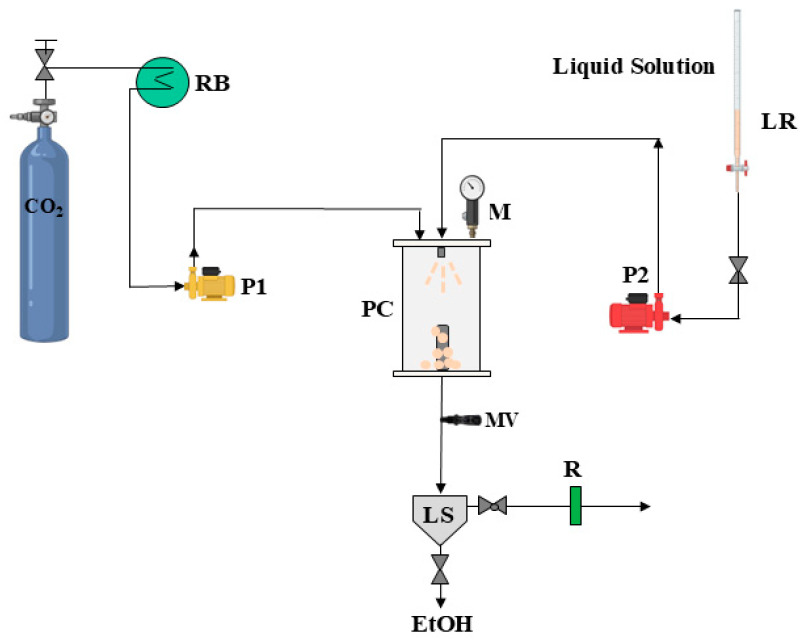
Schematic diagram of the supercritical antisolvent (SAS) laboratory plant, composed of LR: liquid reservoir; LS: liquid separator; M: manometer; MV: micrometric valve; P1, P2: pumps; PC: precipitation chamber, RB: refrigerating bath; and R: rotameter.

The SAS process was used to micronize the Ce(acac)_3_ powder. The SAS plant used for precipitations is schematically reported in Figure 1. The SAS experiment begins when CO_2_ is pumped inside the precipitator to obtain the appropriate pressure. Then, to obtain the quasi-steady state condition of the liquid solution, a certain amount of the liquid solvent is fed inside the chamber. Afterward, once steady-state conditions have been reached, the liquid solution is fed through a 100 µm diameter stainless-steel nozzle. Two high-pressure pumps were employed to feed the CO_2_ and the liquid phase. Once the precipitation is achieved, after a necessary washing step, essential to eliminate the residual solvent, the precipitated particles are collected with a cylindrical porous filter (pores diameter of 0.1 μm). Subsequently, the CO_2_ pump is switched off, and the precipitation chamber is slowly depressurized to atmospheric pressure. In particular, the flow rates of the CO_2_ and liquid used for the SAS experiments were equal to 30 g min^−1^ and 1 mL min^−1^, respectively. Further details regarding the precipitation mechanism have already been documented in previous studies [57].

In this work, for the micronization of Ce(acac)_3_, the conditions optimized in previous work [54] were used (i.e., a temperature equal to 40 °C, a pressure of 150 bar, and a solute total concentration of 5 mg mL^−1^.) Ethanol was used as the solvent. At the end of the experiments, the precipitated powder was collected, characterized, and, after an opportune calcination, used in the photocatalytic tests.

### 2.3. Characterization Methods

A field emission scanning electron microscope (FESEM, mod. LEO 1525, Carl Zeiss SMT AG, Oberkochen, Germany) was employed to examine the morphological characteristics of the samples. The powdered materials were dispersed on a carbon tab to be covered with a thin layer of gold (layer thickness 250 Å) to enhance the conductivity.

The average particle diameters and size distributions of the processed powder were determined by analyzing approximately 500 particles per sample, utilizing the Scan Pro image analysis software v5.0 (Aspire Software International, Ashburn, VA, USA).

Instead, PSDs of the photocatalysts were evaluated by dynamic light scattering analysis, using a Zetasizer Nano ZS (Malvern Instruments Ltd., Worcestershire, UK). Size evaluation analyses were performed in triplicate.

Infrared spectra were recorded using a Fourier Transform (FT-IR) spectrometer (Alpha model, Bruker Optics, Coventry, UK). To prepare the samples, 100 mg of KBr powder was mixed with 1 mg of the sample to obtain an infrared transparent matrix. The scan wavenumber range was 4000–400 cm^−1^.

Ultraviolet-visible diffuse reflectance spectra (UV–Vis DRS) of the samples were acquired using a Perkin Elmer Lambda 35 spectrophotometer (Waltham, MA, USA) equipped with an RSA-PE-20 reflectance spectroscopy accessory (Labsphere Inc., North Sutton, NH, USA). The band gap energies were determined through the Kubelka–Munk function (F(R∞)), by plotting [F(R∞) × hν]^0.5^ as a function of hυ (eV).

Raman spectra were collected at room temperature using a Dispersive MicroRaman spectrometer (Renishaw, Wotton-under-Edge Gloucestershire, UK), with a 514 nm laser, over a Raman shift range of 200–1000 cm^−1^.

X-ray diffraction (XRD) patterns were collected using a Bruker D8 Advance diffractometer equipped with a nickel-filtered Cu-Kα radiation source and a Bragg–Brentano θ-θ geometry. The crystallite sizes were calculated from the Scherrer equation applied to the (111) CeO_2_ crystalline plane.

The Brunauer, Emmett, and Teller (BET) specific surface area (SSA) was determined from dynamic nitrogen adsorption measurements at −196 °C, conducted on a Costech Sorptometer 1042 instrument (Milan, Italy). Before the measurements, the samples were pretreated at 150 °C for 180 min under a helium flow.

### 2.4. Photocatalysts Preparation

Since the SAS process does not lead to the formation of CeO_2_, a subsequent calcination step of the micronized precursor is necessary. A slow calcination process was employed for both samples, increasing the temperature by 2 °C min^−1^ up to the selected calcination temperature (450 °C, 600 °C, or 800 °C), then held at this temperature for two hours [58]. The photocatalytic performance of the catalyst obtained from the processed precursor (CeO_2_-SAS) was compared with that of the catalyst obtained after the calcination of the non-micronized precursor (CeO_2_-NM). The prepared photocatalysts were named:

- CeO_2_ from non-micronized precursor calcined at 450 °C (CeO_2_-NM)
- CeO_2_ from SAS-micronized precursor calcined at 450 °C (CeO_2_-SAS 450 °C)
- CeO_2_ from SAS-micronized precursor calcined at 600 °C (CeO_2_-SAS 600 °C)
- CeO_2_ from SAS-micronized precursor calcined at 800 °C (CeO_2_-SAS 800 °C)

### 2.5. Photocatalytic Experiments

In this study, CAP was used as the target antibiotic for photocatalytic tests under visible light irradiation. The suspension was contained within a cylindrical Pyrex reactor (ID = 3 cm and Volume = 200 mL, Microglass Heim Srl, Naples, Italy) and the irradiation was provided by a visible-LED strip (nominal power: 10 W; emission in the range of 400–800 nm) wrapped around the outer surface of the reactor. The reactor was also equipped with an air distribution system to ensure adequate mixing and avoid the sedimentation of the catalyst at the bottom of the reactor. The initial experiments were carried out to assess the effect of calcination temperature and micronization process on catalyst performance. In this case, 300 mg of catalyst was dispersed in 100 mL of chloramphenicol solution, with an antibiotic concentration of 25 mg L^−1^. The spontaneous pH of the solution was equal to 6.3 during the overall experiments. In subsequent photocatalytic activity tests, photocatalyst dosage and CAP initial concentration were also varied. For all the experiments, the total duration was 300 min, consisting of a 120-min dark phase, necessary to achieve the adsorption–desorption equilibrium on the photocatalyst surface, followed by 180 min under visible light. The samples were collected at regular intervals throughout the experiment, with the first sample representing time zero, taken before the addition of the photocatalyst. A high-performance liquid chromatography (HPLC) system was employed to monitor pollutant concentration over time. The analyses were performed utilizing C18 (Agilent Technologies, Santa Clara, CA, USA) as a stationary phase column, operated at isocratic elution. Following a previous study, a 65/35 *v*/*v* mixture of methanol and bidistilled water was used as a mobile phase, with a flow rate of 1 mL min^−1,^ and detection was performed at a wavelength of 274 nm [59]. The system measured the area under the chromatographic peak corresponding to the retention time characteristic of the target compound which eluted, in this case, at 3.125 min. A calibration process was carried out in advance, generating a calibration curve that was subsequently employed to determine the CAP concentration in the solution.

The following formula was employed for the CAP removal efficiency:

$$\mathrm{CAP}\;\mathrm{removal}\;\mathrm{efficiency}\;(\%)=\left(\frac{C_{0}-C}{C_{0}}\right)*100$$

where C indicates the antibiotic concentration at generic irradiation time (mg L^−1^) while C_0_ is the chloramphenicol concentration after the dark phase (mg L^−1^).

To find a potential mechanism for CAP photocatalytic degradation, a series of experiments were conducted in the presence of scavenger molecules. The reactive substances used were ethylenediaminetetraacetic acid (EDTA), isopropyl alcohol (IPA), and p-benzoquinone (BZQ) for the trapping of holes, hydroxyl radical, and superoxide ions, respectively.

## 3. Results and Discussion

### 3.1. Characterization of Ce(acac)_3_ Micronized Particles Produced by SAS Process

**Figure 2:**
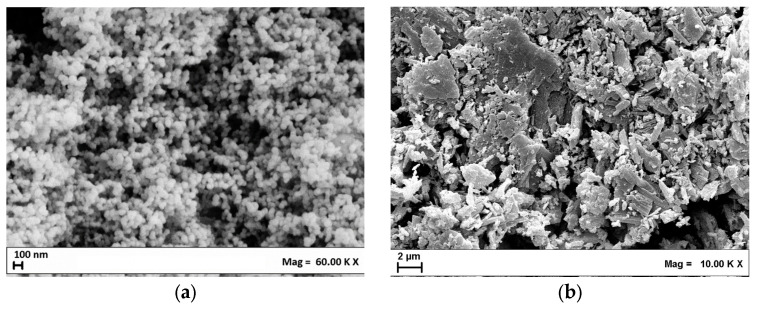
FESEM images of (**a**) Ce(acac)_3_ micronized by SAS process at 150 bar and 40 °C and (**b**) unprocessed Ce(acac)_3_.

The FESEM image of Ce(acac)_3_ produced by the SAS process is shown in Figure 2a, whereas the FESEM image of the unprocessed Ce(acac)_3_ is reported in Figure 2b.

**Figure 3:**
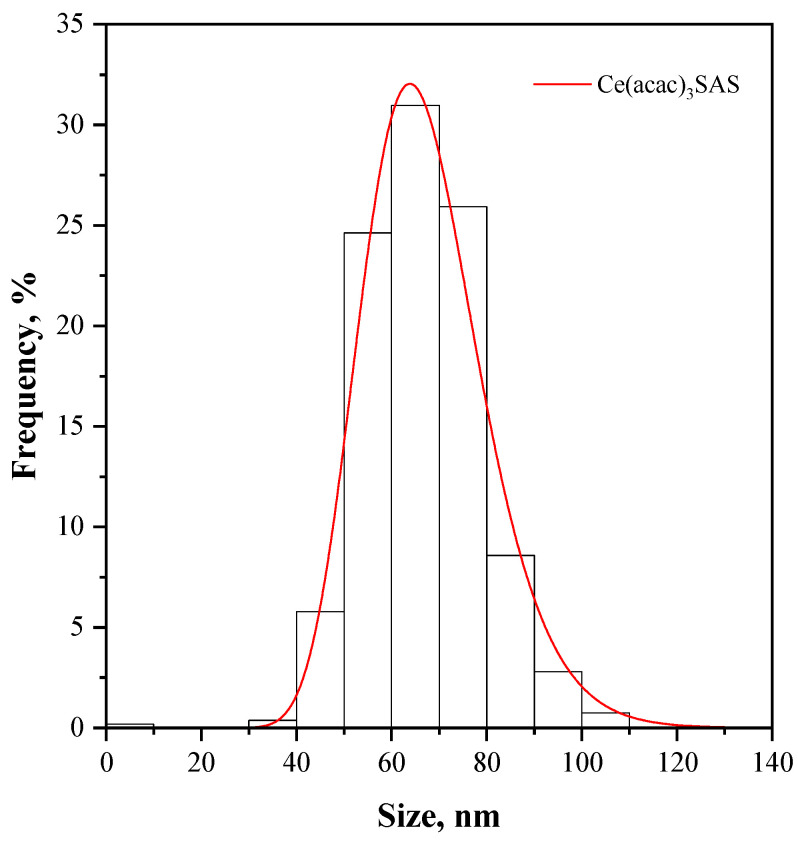
PSD of SAS processed Ce(acac)_3_ (labeled in figure as Ce(acac)_3_SAS).

FESEM images allow us to compare morphologies between the SAS-micronized and the unprocessed sample, revealing a significant change in particle morphology after the SAS process. The unprocessed precursor appears to be made up of large crystals, while the material processed with SAS is precipitated in the form of nanometric particles. Figure 3 shows the particle size distribution of the SAS-micronized sample, obtained through the analysis of the FESEM images with the aid of Sigma Scan Pro 5.0 software. The particles have dimensions lower than 100 nm, with an average size of approximately 65 nm.

**Figure 4:**
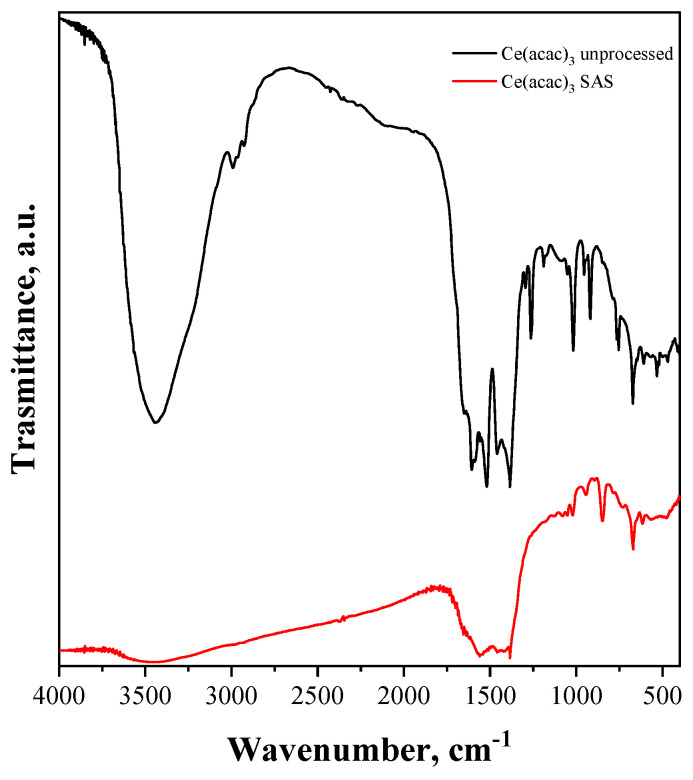
FT-IR spectra of unprocessed and SAS-processed Ce(acac)_3_.

The FT-IR spectra of both samples (reported in Figure 4) exhibit an absorption band at 3500 cm^−1^ attributed to the stretching mode of the adsorbed water. In addition, the absorption band at around 1600 cm^−1^ can be attributed to the OH bending vibration of physically adsorbed water molecules [60]. As already reported in the literature, FT-IR spectroscopy shows that the Ce(acac)_3_ had been modified by the SAS process, because most of the bands of the FT-IR spectrum of the unprocessed Ce(acac)_3_ were different [61].

### 3.2. Characterization of CeO_2_ Particles from Thermal Annealing of Ce(acac)_3_ Micronized Particles

**Figure 5:**
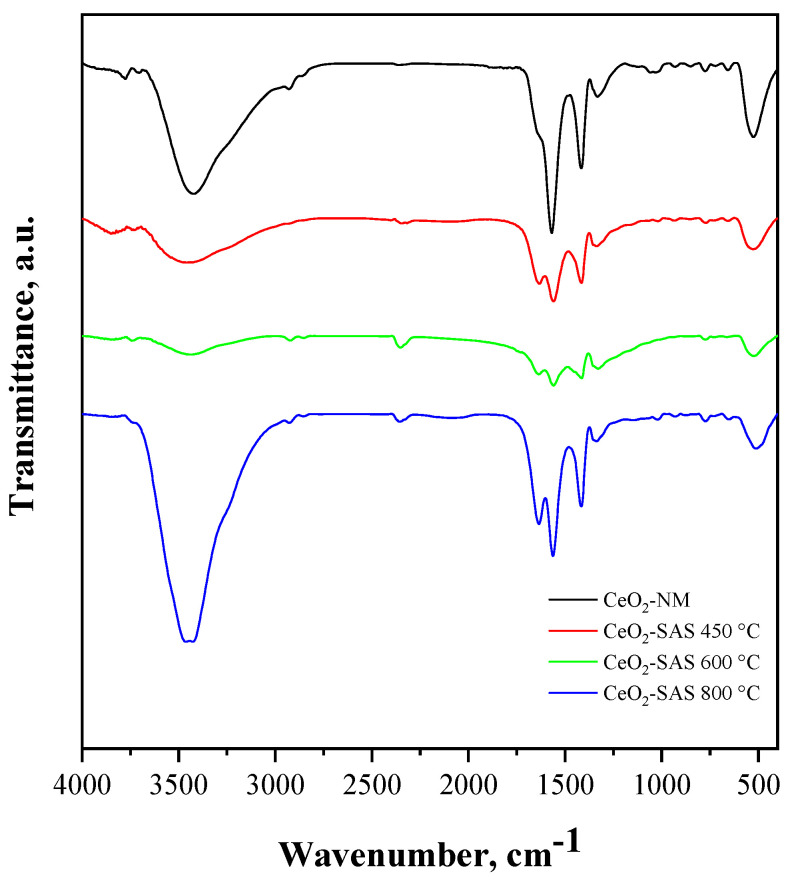
FT-IR spectra of CeO_2_-SAS and CeO_2_-NM photocatalysts.

The photocatalysts obtained after thermal annealing of the non-micronized and SAS-processed precursors were characterized using different techniques (FT-IR spectroscopy, Raman spectroscopy, or UV–Vis diffuse reflectance spectroscopy). In the FT-IR spectra of all the CeO_2_ samples (Figure 5), the widened absorption band in the range of 3000–3750 cm^−1^ can be attributed to the O–H-stretching vibrations of physisorbed H_2_O or to surface Ce–OH groups [62]. The absorption features observed at 1410 cm^−1^ and 1560 cm^−1^ suggest the presence of carbonate-like species formed by the coordination of CO_2_ molecules on the CeO_2_ surface and physically surface-adsorbed CO_2_, respectively [62,63]. In addition, the FT-IR spectra show a band at about 510 cm^−1^ attributed to Ce–O stretching modes, corresponding to the vibrations of the oxygen atoms bonded to cerium in the crystal lattice [64]. Noticeably, the band located at about 1620 cm^−1^, assigned to the bending vibration of the hydroxyl group adsorbed on the surface of the CeO_2_ [64,65], is more prominent for the CeO_2_-SAS samples than for CeO_2_-NM. Since the intensity of this band is associated with the properties of the CeO_2_ surface, such as the presence of oxygen vacancies, which can enhance the adsorption of hydroxyl groups or water molecules [66,67], it can be inferred that all the CeO_2_-SAS samples exhibit a higher concentration of surface oxygen vacancies in comparison to the CeO_2_-NM sample, as corroborated by the Raman spectroscopy findings (*vide infra*).

**Figure 6:**
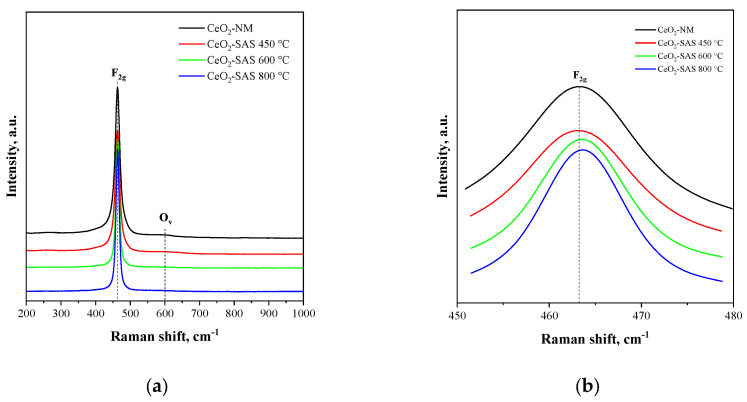
(**a**) Raman spectra for all tested photocatalysts and (**b**) Raman spectra for all tested photocatalysts from 450 cm^−1^ to 480 cm^−1^.

Figure 6a shows the Raman spectra for all the photocatalysts tested in the range of 200–1000 cm^−1^. The main band at 462 cm^−1^ corresponds to the F_2g_ symmetric vibrational mode of the oxygen atoms surrounding the Ce^4+^ ions in the fluorite lattice of pure CeO_2_ [68], whereas, the weak band detected at about 607 cm^−1^ is generally attributed to the presence of oxygen vacancies (O_V_) in the CeO_2_ lattice [69].

In particular, the variation in the intensity of O_v_ bands at 607 cm^−1^ is useful to quantify the transformation from Ce^4+^ to Ce^3+^, and the concentration of oxygen vacancies in CeO_2_ is described using the O_v_/F_2g_ peak intensity ratio [70]. The larger the ratio, the higher the oxygen vacancy concentration is. From Figure 6b, it is possible to observe that with the increase of annealing temperature, the position of the F_2g_ band shifts from 462.8 cm^−1^ at 450 °C to 463.4 cm^−1^ at 600 °C and 463.7 cm^−1^ at 800 °C. This is usually caused by the variation of the bond length, phonon relaxation, or stress in the CeO_2_ nanocrystals [71]. Concurrently, as shown in Table 1, the O_v_/F_2g_ ratio diminishes with increasing temperature, resulting in a lower oxygen vacancy concentration for the CeO_2_-SAS samples synthesized at 600 and 800 °C compared to CeO_2_-SAS 450 °C. However, no significant differences in the O_v_/F_2g_ ratio between CeO_2_ NM and CeO_2_-SAS 450 °C are evident.

**Figure 7:**
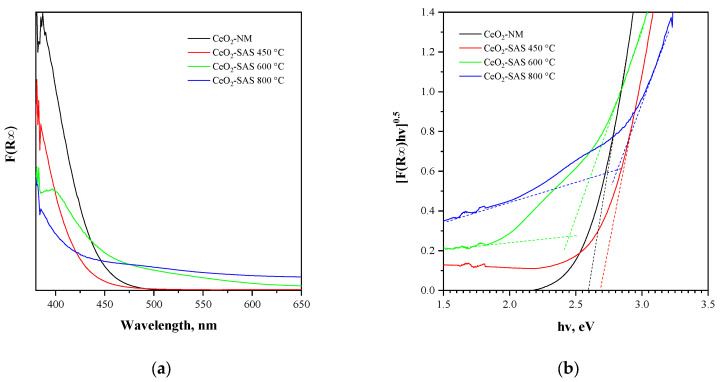
(**a**) F(R∞) vs. wavelength spectra of all the tested photocatalysts and (**b**) a Tauc plot of all the tested photocatalysts.

The data obtained from UV–Vis reflectance spectra (Figure 7a) were used for evaluating the optical band gap energy of the photocatalysts by plotting [F(R_∞_) × hν]^0.5^ vs. hν (Figure 7b). The obtained values are reported inTable 1. For the CeO_2_-SAS samples, at the lowest annealing temperature (450 °C), CeO_2_ remains mostly in its Ce^3^⁺ oxidation state, and oxygen vacancies are more abundant than the CeO_2_-SAS 600 °C and CeO_2_-SAS 800 °C samples (see Raman results). At the highest temperature (800 °C), the oxygen vacancy concentration becomes very low (see Raman results), meaning that CeO_2_ is largely in its Ce^4^⁺ oxidation state. As a result, the band gap energy further increases. However, CeO_2_-NM has a lower optical band gap than the CeO_2_-SAS 450 °C sample. This feature can be explained by considering that, in CeO_2_, the presence of oxygen vacancies facilitates the formation of mixed-valence states of cerium (Ce^3^⁺ and Ce^4^⁺), which contribute to the creation of defect states near the Fermi level [72,73,74]. The higher concentration of oxygen vacancies in the CeO_2_-NM and CeO_2_-SAS 450 °C samples (Table 1) may result in fewer defect states, leading to a relatively narrower gap for electron excitation. Additionally, it must be considered that oxygen vacancies can modify the crystal lattice and introduce strain, which also affects the band structure [75].

**Figure 8:**
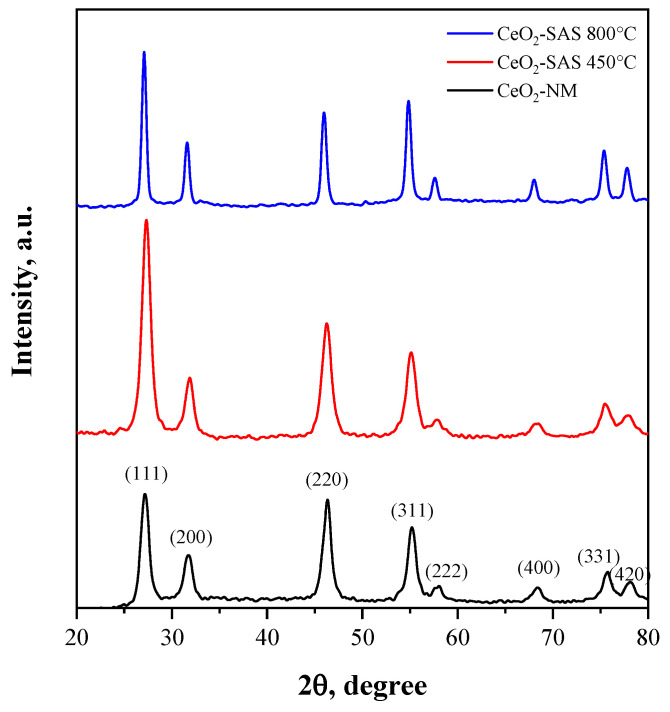
XRD patterns of CeO_2_-NM, CeO_2_-SAS 450 °C, and CeO_2_-SAS 800 °C.

The structural properties of CeO_2_-NM, CeO_2_-SAS 450 °C, and CeO_2_-SAS 800 °C were analyzed through X-ray diffraction (XRD) patterns (Figure 8) and corroborated by crystallite size calculations using the Scherrer equation and specific surface area measurements (Table 2). The observed diffraction peaks can be indexed to the fluorite cubic structure of cerium dioxide (CeO_2_) [76,77], in agreement with the standard JCPDS for CeO_2_ (card n° 34-0394). Prominent peaks at 2θ values of approximately 28.5°, 33.0°, 47.5°, 56.5°, 59.1°, 69.5°, and 76.8° are assigned to the (111), (200), (220), (311), (222), (400), and (420) planes, respectively, confirming the cubic fluorite phase. Significant differences in peak intensity and width were observed among the samples, reflecting variations in crystallinity and crystallite size. CeO_2_-NM displays the broadest peaks, indicative of the smallest crystallite size (8 nm, as calculated using the Scherrer equation) and the highest specific surface area (43 m^2^·g⁻^1^). For CeO_2_-SAS 450 °C, the XRD peaks are slightly sharper, corresponding to a moderate increase in crystallite size to 9 nm and a decrease in specific surface area to 34 m^2^·g⁻^1^. This indicates partial grain growth during calcination at 450 °C while preserving a relatively high surface area. The CeO_2_-SAS 800 °C sample shows the sharpest XRD peaks, reflecting the largest crystallite size (17 nm) and the lowest specific surface area (8 m^2^·g⁻^1^). This behavior is attributed to significant grain growth and sintering during high-temperature calcination, which enhances crystallinity while substantially reducing surface area. The interplay between crystallite size and specific surface area highlights the critical role of thermal treatment in determining the structural and morphological characteristics of CeO_2_. Higher calcination temperatures promote crystallinity and reduce lattice strain, as evidenced by the sharper diffraction peaks, but also cause sintering and a corresponding decrease in surface area.

### 3.3. Photocatalytic Activity Results

**Figure 9:**
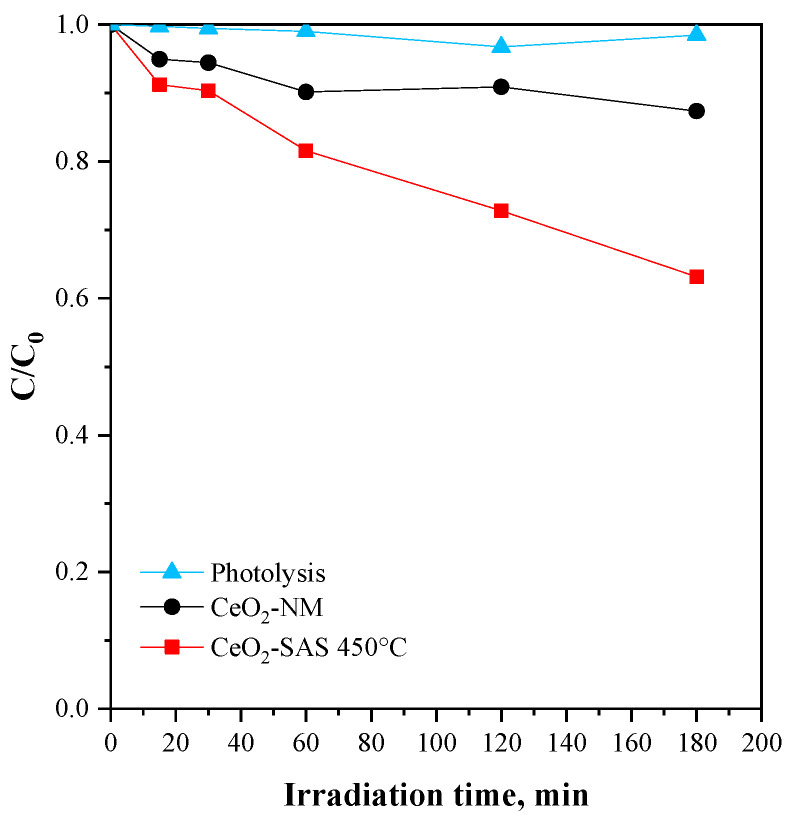
Degradation of the chloramphenicol under visible light on CeO_2_-NM and CeO_2_-SAS 450 °C compared to the photolysis test.

The initial experiments aimed to compare the performance of the catalyst obtained from the micronized precursor with that of the catalyst derived from the untreated precursor. In this case, 300 mg of catalyst was dispersed in 100 mL of chloramphenicol solution, with an antibiotic concentration of 25 mg L^−1^. The graph reported in Figure 9 illustrates the relative concentration of chloramphenicol (C/C_0_) as a function of irradiation time. The dataset compares photolysis, CeO_2_-NM, and CeO_2_-SAS 450 °C over a period of 180 min of treatment time. The photolysis process shows negligible degradation, as the relative concentration remained close to 1 throughout the experiment. Conversely, the CeO_2_-NM sample exhibits moderate photocatalytic activity, reaching approximately 10% of CAP degradation after 180 min of irradiation. Notably, the CeO_2_-SAS 450 °C catalyst demonstrates the highest photocatalytic activity since, at the end of the irradiation period, the CAP degradation was 37%, indicating a superior performance compared to the CeO_2_-NM photocatalyst.

This outcome may result from the difference in morphology and size between the two photocatalysts. For this reason, the particle size was analyzed and the results for hydrodynamic diameters in solution (deionized water) are presented in Table 3. For CeO_2_-SAS 450 °C, the hydrodynamic diameter was observed to be 313.8 nm while the hydrodynamic diameter increased to 621.6 nm for CeO_2_-NM.

**Figure 10:**
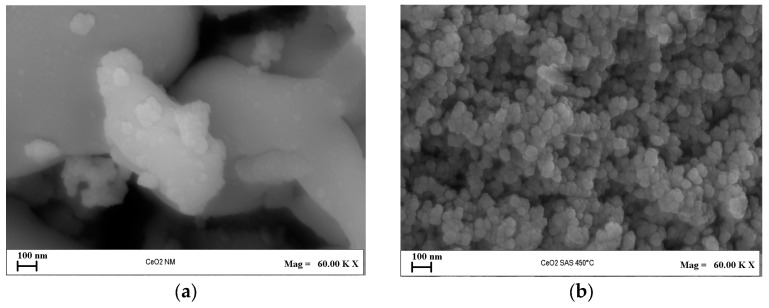
FESEM images of (**a**) CeO_2_-NM and (**b**) CeO_2_-SAS 450 °C.

From Figure 10, it is possible to observe that the CeO_2_-SAS 450 °C sample shows predominantly spherical nanoparticles that are non-uniform in size. In contrast, the CeO_2_-NM photocatalyst, obtained from the non-micronized precursor, shows a compact and non-micrometric structure.

A higher particle size value indicates that the CeO_2_-NM particles tend to agglomerate, forming larger clusters when dispersed in water. This aggregation limits the surface available for the contaminant, potentially affecting the efficiency of the catalytic process [78].

#### 3.3.1. Effect of Annealing Temperature on CeO_2_-SAS Photocatalysts

**Figure 11:**
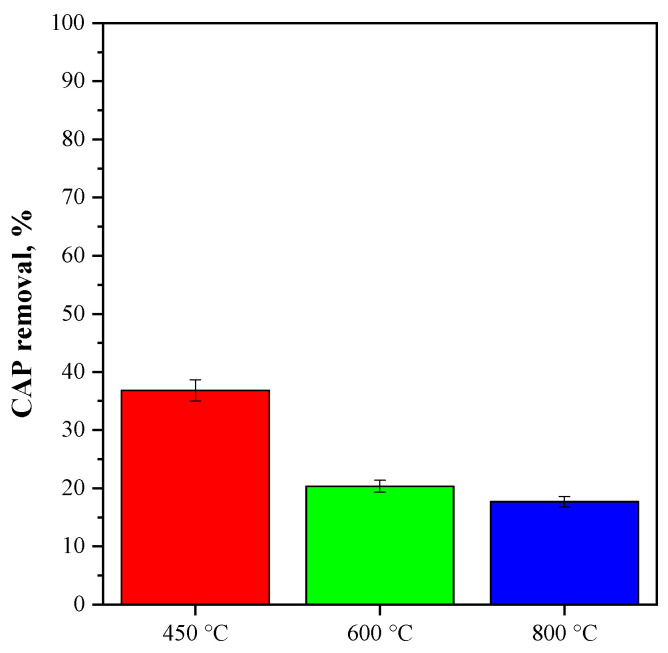
Percentage of CAP removal after 180 min of visible light irradiation in the presence of CeO_2_-SAS annealed at different temperatures. Photocatalyst dosage: 3 g L^−1^.

The effect of the annealing temperature on the photocatalytic activity was also evaluated in the CeO_2_-SAS samples since it can affect the CAP degradation performance. The results obtained are reported in Figure 11.

The CeO_2_-SAS 450 °C photocatalyst exhibited superior photocatalytic activity compared to the CeO_2_-SAS 600 °C and CeO_2_-SAS 800 °C photocatalysts. In detail, a CAP removal of 37% was obtained for the sample annealed at 450 °C, and it was reduced to 20 and 17% for the CeO_2_-SAS samples annealed at 600 and 800 °C, respectively. This result could be due to CeO_2_-SAS 450 °C’s higher concentration of oxygen vacancies (as evinced both from the FT-IR and Raman results), which significantly enhances the photocatalytic properties. More in detail, at a 450 °C annealing temperature, CeO_2_-SAS retained a higher concentration of oxygen vacancies because the formation of vacancies is favored at lower temperatures, where the oxygen loss from the lattice is less pronounced [79,80,81]. These oxygen vacancies act as shallow traps for electrons, creating a high density of reactive species, like superoxide radicals, that can degrade the target pollutant more effectively under light irradiation [82,83,84]. Indeed, the photocatalytic tests in the presence of scavenger molecules evidenced that the superoxide radical is the main reactive oxygen species involved in the CAP degradation mechanism (*vide infra*). In contrast, higher annealing temperatures (600 °C and 800 °C) led to a reduction in the number of oxygen vacancies, as the elevated temperatures promote the reordering of oxygen atoms in the CeO_2_ lattice, reducing the semiconductor defect density [81,85]. Consequently, the photocatalytic activity of CeO_2_-SAS 600 °C and CeO_2_-SAS 800 °C was lower because the reduced number of oxygen vacancies led to less efficient electron–hole separation and fewer active sites for the photocatalytic reaction.

#### 3.3.2. Effect of CeO_2_-SAS 450 °C Dosage on CAP Photocatalytic Degradation

**Figure 12:**
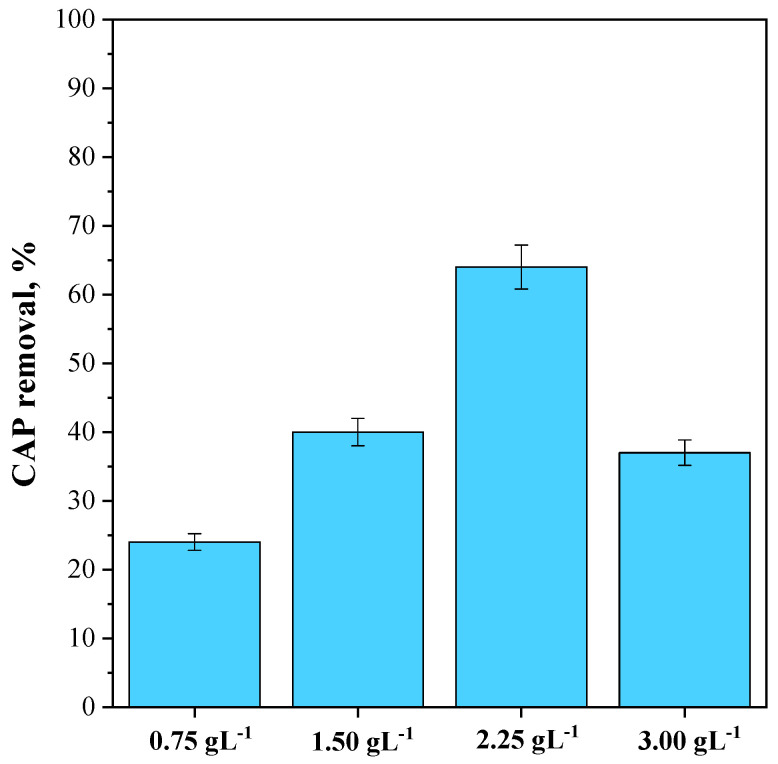
Effect of CeO_2_-SAS 450 °C dosage on CAP photocatalytic degradation.

To investigate the impact of photocatalyst dosage, photocatalytic experiments were conducted using a fixed CAP concentration equal to 25 mg L^−1^ and varying the CeO_2_-SAS 450 °C dosage from 0.75 g L^−1^ to 3 g L^−1^. The results obtained are reported in Figure 12. It was noted that at the lowest investigated photocatalyst dosage (0.75 g L^−1^), the CAP degradation was only 24% after 180 min of visible light irradiation. However, by increasing the photocatalyst dosage to 1.50 g L^−1^, the CAP degradation increased to 40%, reaching the maximum value of 64% when the photocatalyst dosage was equal to 2.25 g L^−1^. Furthermore, at a higher dosage of 3 g L^−1^, the CAP degradation decreased to 37%.

At lower CeO_2_-SAS 450 °C dosages (in the range of 0.75–2.25 g L^−1^), the photocatalytic performance increased as more active sites on the photocatalyst surface became available to absorb visible light and interact with the CAP molecules, leading to an increase in the degradation efficiency [30]. However, when the CeO_2_-SAS 450 °C dosage was increased to 3 g L^−1^, the photocatalytic efficiency declined because the excess of photocatalyst particles turbid the suspension, reducing the effective exposure of each particle to light [86,87].

#### 3.3.3. Influence of CAP Initial Concentration on CeO_2_-SAS 450 °C Photocatalytic Performance

**Figure 13:**
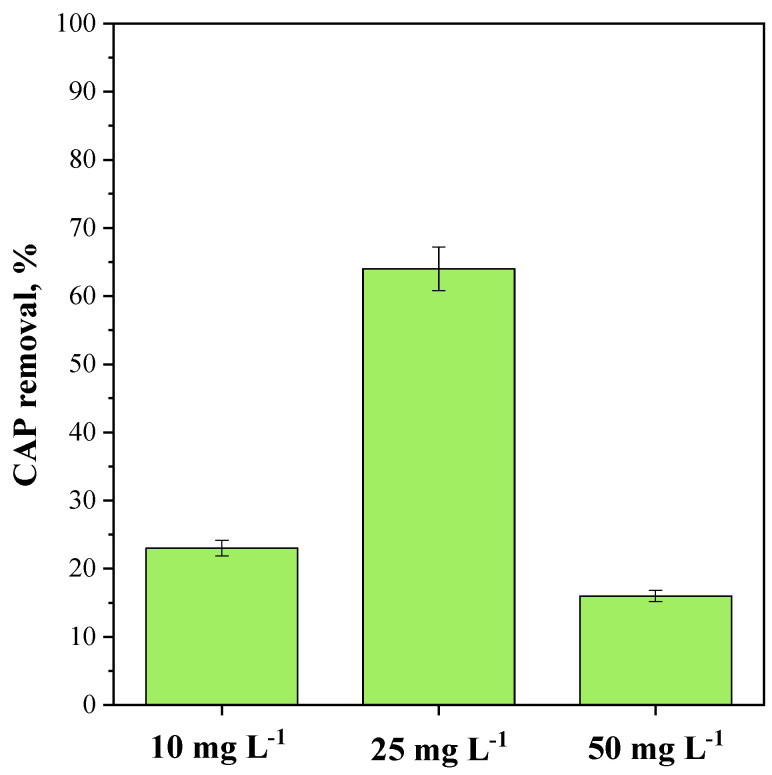
Effect of the CAP initial concentration on the photocatalytic performance of CeO_2_-SAS 450 °C after 180 min of visible light irradiation. Photocatalyst dosage: 2.25 g L^−1^.

To investigate the impact of the CAP initial concentration on the photocatalytic degradation efficiency, experiments were carried out using the optimized CeO_2_-SAS 450 °C dosage (2.25 g L^−1^) and varying the antibiotic concentration from 10 mg L^−1^ to 50 mg L^−1^. Figure 13 illustrates that the CAP removal rose with an increasing initial concentration within the 10–25 mg L^−1^ range but declined at a higher CAP concentration (50 mg L^−1^). Previous studies have indicated that as pollutant concentration increases, the demand for reactive species to degrade the antibiotic also increases. Nevertheless, under fixed conditions of light intensity, photocatalyst loading, and irradiation time, the generation of radical species remains stable. Consequently, the available quantity of reactive species can become insufficient to degrade the pollutant effectively [88]. Moreover, at high initial CAP concentration, the possible accumulation of reaction intermediates can hinder the photocatalytic process. Indeed, these intermediates, which are generated during the partial degradation of CAP, may compete for active sites on the photocatalyst surface, diminishing the accessibility of these sites for further degradation of the parent compound [89]. This competition can result in decreased photocatalytic activity, as the generated intermediates may adsorb onto the photocatalyst surface, restricting the adsorption of additional CAP molecules from the liquid phase.

#### 3.3.4. Role of Reactive Oxygen Species (ROS) on CAP Photodegradation Catalyzed by CeO_2_-SAS 450 °C

**Figure 14:**
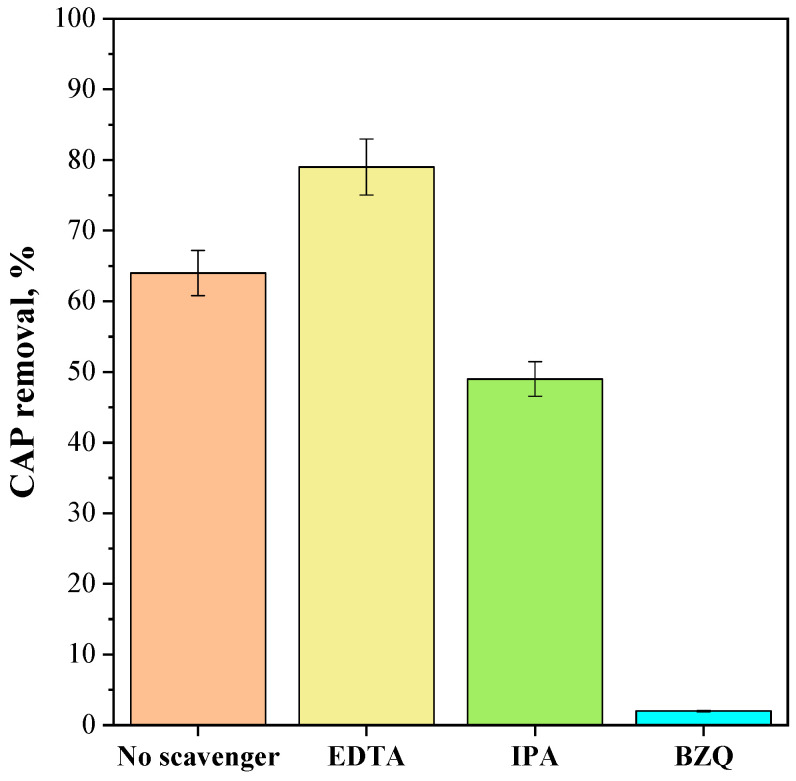
Effect of the scavengers on the photocatalytic degradation of CAP after 180 min of visible light irradiation in the presence of the CeO_2_-SAS 450 °C photocatalyst. Photocatalyst dosage: 2.25 g L^−1^; initial CAP concentration: 25 mg L^−1^.

Further photocatalytic experiments were carried out to examine the potential impact of reactive oxygen species (ROS) on the degradation of CAP in the presence of the CeO_2_-SAS 450 °C photocatalyst. Specifically, photocatalytic degradation tests were performed using EDTA, benzoquinone (BZQ), and isopropyl alcohol (IPA) as scavengers for holes [90], superoxide [91,92], and hydroxyl radicals [93], respectively. To this end, under optimal reaction conditions (photocatalyst dosage: 2.25 g L^−1^; initial CAP concentration: 25 mg L^−1^), EDTA (at a concentration of 10 mmol L^−1^), BQ (at a concentration of 1 μmol L^−1^), and IPA (at a concentration of 10 mmol L^−1^) were added to the CAP solution. Figure 14 illustrates the effects of the scavenger’s presence on photocatalytic performances. The presence of IPA, BQ, and EDTA affected the photocatalytic degradation of CAP. In particular, IPA slightly decreased CAP removal, while BQ nearly completely suppressed the photocatalytic reaction. These results suggest that superoxide radicals are the primary reactive oxygen species responsible for CAP degradation, followed by hydroxyl radicals. On the other hand, in the presence of EDTA, the degradation ability of the catalyst increased. It could be argued that when EDTA traps holes in the semiconductor valence band, electron–hole pair separation is enhanced, leading to improved photocatalytic activity. This is because more electrons in the photocatalyst conduction band are available to reduce molecular oxygen into superoxide radicals. Overall, the test with EDTA further corroborates the crucial role of superoxide radicals in CAP degradation, aligning with the Raman results indicating the highest oxygen vacancy concentration in the CeO_2_-SAS 450 °C photocatalyst.

#### 3.3.5. Stability Tests on CeO_2_-SAS 450 °C Photocatalyst

**Figure 15:**
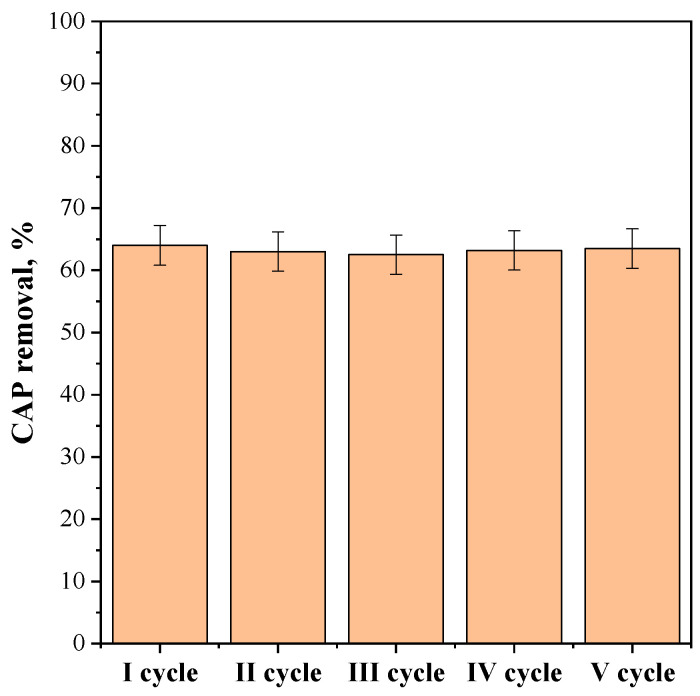
Recyclability tests on CeO_2_-SAS 450 °C photocatalyst. Photocatalyst dosage: 2.25 g L^−1^; initial CAP concentration: 25 mg L^−1^.

Additional photocatalytic tests were carried out to evaluate the stability of the CeO_2_-SAS 450 °C photocatalyst that displayed the highest photocatalytic activity. Specifically, the experiments were performed using a catalyst dosage of 2.25 g L^−1^, with an initial CAP concentration of 25 mg L^−1^. The photocatalyst was recovered from the suspension after each test, cleaned with distilled water, and reused again in a subsequent test with the same operating conditions. The results, after five reuse cycles, are reported in Figure 15, in terms of CAP removal after 180 min of visible light irradiation. The obtained results demonstrated the stability of the photocatalyst as its photocatalytic efficiency remained consistent throughout five consecutive reuse cycles.

## 4. Conclusions

In this study, CeO_2_ photocatalysts were synthesized via the supercritical antisolvent (SAS) technique and subsequently evaluated for their photocatalytic degradation of chloramphenicol. The SAS method enabled the production of CeO_2_ nanoparticles with enhanced photocatalytic activity compared to those derived from the unprocessed precursor. The influence of annealing temperature was also investigated, revealing that temperatures exceeding 450 °C led to a decrease in CeO_2_ oxygen vacancies, a widening of the band gap, and consequently, diminished photocatalytic performance. Further photocatalytic experiments were conducted to explore the impact of the chloramphenicol initial concentration and photocatalyst dosage. Specifically, employing a catalyst dosage of 2.25 g L^−1^ and an antibiotic concentration of 25 mg L^−1^, a degradation efficiency of 64% was achieved. Various scavengers were utilized to propose a plausible degradation mechanism, indicating that the superoxide radical is the primary species responsible for chloramphenicol degradation. Moreover, the optimized photocatalyst exhibited excellent recyclability and reusability over five consecutive reuse cycles. This preliminary study aimed to assess the feasibility of using the SAS method to develop novel CeO_2_-based photocatalysts with improved visible light absorption and catalytic activity for future research.

## Figures and Tables

**Table 1:** O_v_/F_2g_ intensity ratios and band gap of all tested photocatalysts.

Photocatalyst	O_v_/F_2g_ (-)	Band Gap (eV)
CeO_2_-NM	0.0268	2.60
CeO_2_-SAS 450 °C	0.0280	2.70
CeO_2_-SAS 600 °C	0.0120	2.45
CeO_2_-SAS 800 °C	0.0096	2.80

**Table 2:** Crystallite size and specific surface area (SSA) of CeO_2_-NM, CeO_2_-SAS 450 °C, and CeO_2_-SAS 800 °C.

Photocatalyst	Crystallite Size (nm)	SSA (m^2^ g^−1^)
CeO_2_-NM	8	43
CeO_2_-SAS 450 °C	9	34
CeO_2_-SAS 800 °C	17	8

**Table 3:** Hydrodynamic diameter and polydispersity index of CeO_2_-NM and CeO_2_-SAS 450 °C.

Photocatalyst	D_h_ (nm)	PdI (-)
CeO_2_-NM	621.6	0.463
CeO_2_-SAS 450 °C	313.8	0.337

## Data Availability

The data presented in this study are available on request from the corresponding authors.
